# Molecular and Functional Characterization of MobK Protein—A Novel-Type Relaxase Involved in Mobilization for Conjugational Transfer of *Klebsiella pneumoniae* Plasmid pIGRK

**DOI:** 10.3390/ijms22105152

**Published:** 2021-05-13

**Authors:** Katarzyna Paulina Nowak, Agnieszka Sobolewska-Ruta, Agata Jagiełło, Anna Bierczyńska-Krzysik, Piotr Kierył, Paweł Wawrzyniak

**Affiliations:** 1 Institute of Biochemistry and Biophysics, Polish Academy of Sciences, Pawińskiego 5a, 02-106 Warsaw, Poland; kpnowak@ibb.waw.pl; 2 Department of Biomedical Technology, Cosmetics Chemicals and Electrochemistry, Łukasiewicz Research Network—Industrial Chemistry Institute, Rydygiera 8, 01-793 Warsaw, Poland; sobolewska@iba.waw.pl (A.S.-R.); agata.klaudia.jagiello@gmail.com (A.J.); anna.krzysik@scopefluidics.com (A.B.-K.); kierylp@iba.waw.pl (P.K.); 3 Central Forensic Laboratory of the Police, Biology Department, Iwicka 14, 00-735 Warsaw, Poland; 4 Curiosity Diagnostics Sp. z o.o., Duchnicka 3, Building 16, Entrance A, 01-796 Warsaw, Poland

**Keywords:** horizontal gene transfer, conjugation, mobile genetic elements, Mob, relaxase, tyrosine recombinase, MobK, pIGRK, *Klebsiella pneumoniae*, G-quadruplex

## Abstract

Conjugation, besides transformation and transduction, is one of the main mechanisms of horizontal transmission of genetic information among bacteria. Conjugational transfer, due to its essential role in shaping bacterial genomes and spreading of antibiotics resistance genes, has been widely studied for more than 70 years. However, new and intriguing facts concerning the molecular basis of this process are still being revealed. Most recently, a novel family of conjugative relaxases (Mob proteins) was distinguished. The characteristic feature of these proteins is that they are not related to any of Mobs described so far. Instead of this, they share significant similarity to tyrosine recombinases. In this study MobK—a tyrosine recombinase-like Mob protein, encoded by pIGRK cryptic plasmid from the *Klebsiella pneumoniae* clinical strain, was characterized. This study revealed that MobK is a site-specific nuclease and its relaxase activity is dependent on both a conserved catalytic tyrosine residue (Y^179^) that is characteristic of tyrosine recombinases and the presence of Mg^2+^ divalent cations. The pIGRK minimal origin of transfer sequence (*oriT*) was also characterized. This is one of the first reports presenting tyrosine recombinase-like conjugative relaxase protein. It also demonstrates that MobK is a convenient model for studying this new protein family.

## 1. Introduction

Conjugational transfer (CT) is one of the main factors promoting DNA mobility and genetic diversity in a bacterial world [1,2]. Different mobile genetic elements, like plasmids, integrative and conjugative elements (ICEs) and integrative and mobilizable elements (IMEs), can transfer their genomes via CT from donor to recipient bacterial cell [3]. A significant consequence of CT is the spreading of antibiotic resistance (AR) among pathogenic bacterial strains [4,5,6,7]. For these reasons, CT has been widely studied and extensive knowledge about the molecular bases of this process has been gained [3,8,9,10,11]. Thus, innovatory strategies for preventing CT-dependent AR spread have been proposed [12]. They rely on the use of specific inhibitors blocking each component of a CT system protein machinery [12,13,14,15,16,17].

In general, there are three basic elements indispensable for DNA transfer from donor to recipient cell in *Proteobacteria* [3]: (i) in cis-acting origin of transfer sequence (*oriT*), (ii) conjugative relaxase Mob (mobilization protein) and (iii) type IV secretion system (T4SS). Mob introduces a strand- and site-specific nick in the *oriT* sequence and initiates rolling circle replication (RCR). A DNA-Mob complex, relaxosome, is recognized by type IV coupling protein (T4CP) and is introduced to the T4SS located in a cell envelope. T4SS mediates the transfer of a single-stranded DNA (ssDNA) to a recipient cell. While some mobile elements possess complete CT modules (conjugal plasmids, ICEs), others only contain the *oriT* sequence and *mob* gene encoding relaxase—Mob protein or even the orphan *oriT* sequence (mobilizable plasmids and IMEs) and their CT depend on the presence of conjugal apparatus in a bacterial cell [3].

Mob proteins are the most specific enzymes and as such, are a diverse group of CT enzymes. Currently, Mobs are classified into eight families [18]. Four of them (MOB_F_, MOB_Q_, MOB_P_ and MOB_V_ families) are phylogenetically related. They belong to HUH nucleases, enzymes with a conserved motif consisting of three amino acid residues (H-histidine, U-hydrophobic residue, H-histidine) responsible for divalent cations binding [19]. Mobs from the MOB_H_ family exhibit some sequence similarities to HUH proteins, but there are no crystallographic data to confirm their structural similarity [19,20]. MOB_C_ proteins are related to PD-(D/E)XK restriction enzymes [21]. Representatives of the MOB_T_ family belong to Rep_*trans* nucleases [22]. A common feature of listed enzymes is a generation of single-strand cleavage in an *oriT* sequence [3,23]. Mobs remain covalently attached to the DNA 5′-end (except MOB_C_ proteins [21]), leading to initiation of RCR replication from free 3′-OH DNA end. For a review of this outcome, see [3]. 

Recently, a new group of Mobs was distinguished [18]. They are not related to any of the MOB families described so far. Surprisingly, they exhibit high similarity to tyrosine recombinases (TRs) [24]. The mechanism of action for TR-like Mobs remains a great mystery, as there is limited data from functional analysis of these proteins. So far, only three TR-like Mob proteins were described: TcpM from *Clostridium perfringens* pCW3 conjugal plasmid [24,25], MpsA from *Salmonella* sp. IME element SGI1 [26] and MobK—an archetype of TR-like Mobs (initially named Int) from *Klebsiella pneumoniae* pIGRK mobilizable plasmid [27]. 

In our previous study, we revealed that, against the bioinformatic predictions, pIGRK carries a functional mobilization (MOB) module [27]. This atypical MOB module is composed of: (i) *int* gene encoding relaxase protein (here renamed to *mobK*); (ii) and the 456 bp DNA region containing an *oriT* sequence (here named oriT1) located just upstream of the *int*/*mobK* gene. Another intriguing feature of the pIGRK MOB module is that it successfully cooperates with non-related T4SS machinery from plasmid RP4. Both the in trans-acting Int/MobK relaxase protein and in cis-acting oriT1 sequence are sufficient for DNA transfer from the *Escherichia coli* donor cell expressing RP4 T4SS genes to the recipient cell [27]. A similarity search, performed using the BLASTP program, showed that the pIGRK relaxase protein shares significant amino acid sequence similarity to tyrosine recombinases containing DNA breaking-rejoining *C*- terminal catalytic domain (DNA_BRE_C), e.g., bacteriophage integrases (for this reason, MobK was initially designated as Int) [27]. It was the first report suggesting that there is a new group of Mob proteins resembling TRs enzymes. 

In this paper, results of detailed in vivo and in vitro studies on MobK protein and its cognate *oriT* sequence are presented.

## 2. Results

### 2.1. Comparative Analysis of Amino Acid Sequences of MobK and Other TR—Like Relaxases

**Figure 1:**
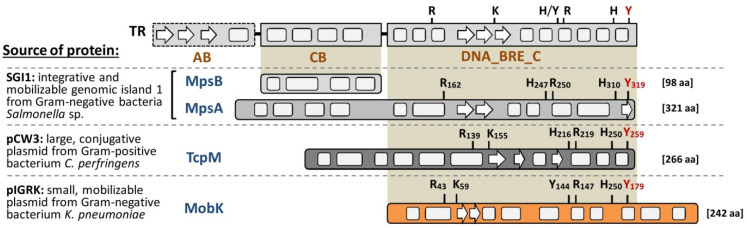
Schematic representation of tyrosine recombinase-like (TR-like) conjugative relaxase’s amino acid sequences. MpsA and MpsB, TcpM as well as MobK sequences, were compared with consensus of tyrosine recombinase (TR) protein [29]: AB—*N*-terminal arm DNA recognizing domain (present only in some TRs); CB—core DNA binding domain; DNA_BRE_C, DNA breaking-rejoining enzymes, *C*-terminal catalytic domain with catalytic pentad [RK(H/Y)RH] and tyrosine nucleophile (marked in red); white arrows and gray rectangles are *β*- strands and *α*- helices, respectively (in silico predicted using JPred4 [30]).

Despite the significant similarity to TR enzymes amino acid sequences of MobK (acc. no. AAS55463.1) and other, recently described, TR-like Mob proteins TcpM (acc. no. ABC96296.1) and MpsA (acc no. AAK02039.1) differ from each other. The lack of significant similarity between TR-like Mobs is not surprising, since in general, the amino acid sequence conservation among TRs is also weak [28,29]. However, TR-like Mob proteins in silico predicted secondary structures and the presence of conserved residues: (i) the tyrosine nucleophile; and (ii) the catalytic RK(H/Y)YRH pentad correspond to DNA_BRE_C catalytic domain of TR enzymes (Figure 1). 

Whole TRs are composed basically of two domains. Besides the DNA_BRE_C catalytic domain, they are obligatorily equipped with the core DNA binding domain (CB). A certain group of TRs also has an additional element: *N*-terminal arm-binding domain (AB). In contrast to known TRs, MobK, TcpM and MpsA are deprived of AB and CB DNA binding domains. Instead of this, both TR-like Mob proteins possess diverse, additional sequence elements on the *N*- or *C*-terminal end (Figure 1). In the case of MpsA, individual accessory protein MpsB (acc. no. AAK38397.1) was identified [26]. It poses an alpha-helical structure and resembles the CB domain of bacteriophage TR integrases. Both MpsA and MpsB are indispensable for CT and MpsA most likely binds DNA through MpsB [23]. No *mpsB* equivalent genes were identified in pIGRK and pCW3, suggesting that MobK and TcpM bind DNA differently. Cryptons, a group of fungal transposable elements (TE), encode atypical TR enzymes with a *C*-terminal alpha-helical DNA binding domain resembling fungal transcriptional regulators [31]. In MobK, two additional alpha-helices are present in the *C*-terminal part of the protein (Figure 1); however, no sequence similarity was identified between crypton TR and MobK (data not shown).

### 2.2. Tyrosine Residue (Y^179^) Is Crucial for the MobK Relaxase Activity 

**Figure 2:**
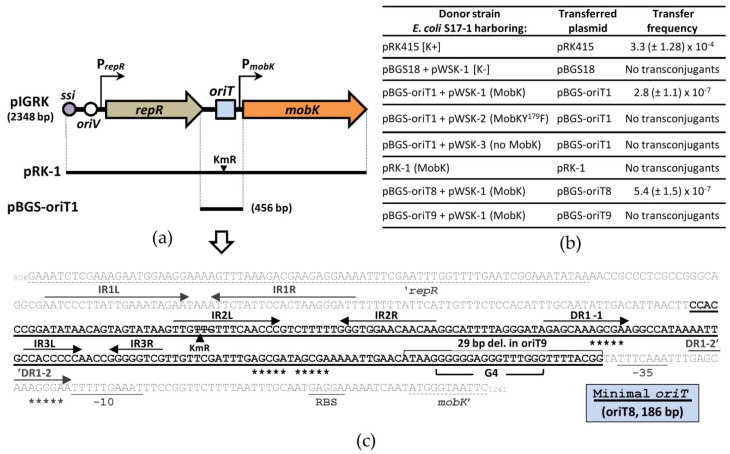
Functional analysis of the components of the pIGRK MOB module. (**a**) Genetic organization of pIGRK. Conjugal transfer origin (*oriT*) and relaxase gene (*mobK*), as well as vegetative replication origin (*oriV*), replication initiator gene (*repR*) and single-strand initiation sequence (*ssi*) are indicated [32]. Promoters of *mobK* (P*_mobK_*) and *repR* (P*_repR_*) genes are marked as black arrows [32]. Black lines represent the DNA fragment of pIGRK and its mutated version with the insertion of a kanamycin resistance cassette (KmR). (**b**) Mutational analysis of pIGRK mobilization system. Transfer frequency is presented as the number of transconjugants per donor cell. As a positive control pRK415 plasmid, containing RK2 *oriT* sequence was used. (**c**) Nucleotide sequence of oriT1 region cloned in pBGS-oriT1. Minimal *oriT* (oriT8 fragment cloned in pBGS-oriT8) is marked in bold black font with solid line underlined. Sequences of the *C*-terminal part of *repR* gene and the *N*-terminal part of *mobK* gene are underlined with dotted lines. Regulatory sequences of *mobK* gene: in silico predicted promoter region (using BPROM [33]) as well as RBS, ribosome binding site are indicated by a wavy line. DNA bases of four 5′-AGCGA-3′ motifs are indicated by asterisks. IR, inverted repeats; DR, direct repeats; DNA bases replaced by kanamycin resistance cassette (KmR) insertion in pRK-1. Putative G—quadruplex DNA (G4), predicted using G4IPDB [34] as well as bases deleted in pBGS-oriT9 are presented.

To verify the role of putative nucleophile Y^179^ in the MobK mobilization function, a previously constructed two-plasmids system [27] was used. A pBGS18 with cloned 456-bp pIGRK fragment (pBGS-oriT1) was utilized as a mobilizable plasmid (Figure 2a) and pWSK-2 served as a source of MobKY^179^F protein (Supplementary Figure S1 and Table S1). As controls, pWSK-1 (source of native MobK) and pWSK-3 (lack of MobK) were used (Supplementary Figure S1 and Table S1). Transconjugants were obtained only when native MobK protein was derived in trans (Figure 2b). This proves that Y^179^ in silico predicted as a catalytic residue (based on MobK similarity to TRs) is critical for the activity of MobK in vivo.

### 2.3. MobK Interacts Specifically with oriT1 Region of pIGRK In Vitro

**Figure 3:**
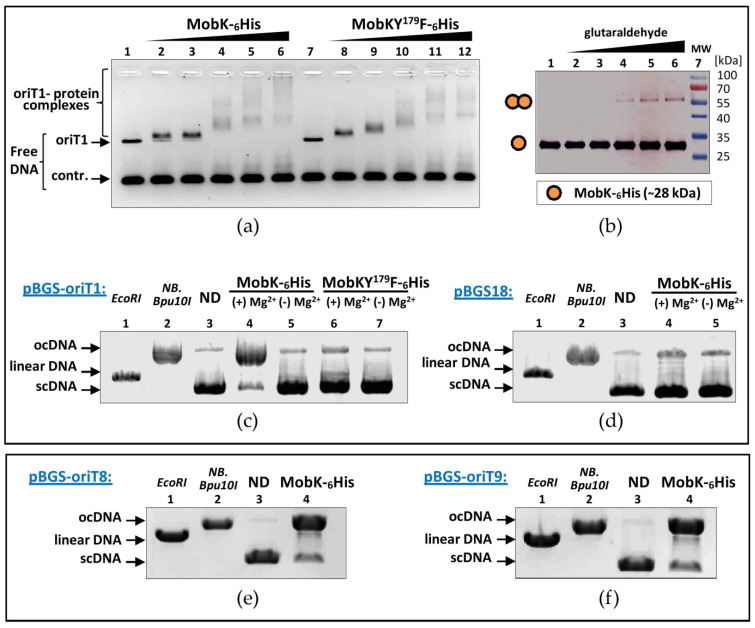
Determination of recombinant MobK protein activities in vitro. (**a**) Electrophoretic mobility shift assay (EMSA), binding of MobK-_6_His and MobKY^179^F-_6_His to fluorescein (FAM)-labeled 592 bp DNA fragment containing pIGRK *oriT*; control 136 bp DNA fragment of pBGS18. Lanes (1) and (7): no protein added, lanes (2–6) and (8–12): samples with increasing amount of protein. (**b**) Dimers formation of MobK-_6_His molecules determined using glutaraldehyde cross-linking, lanes:, (1)—MobK-_6_His incubated without glutaraldehyde, (2–6)—MobK-_6_His incubated with increasing concentration of glutaraldehyde, (7)—protein molecular-weight size marker. (**c**,**d**) Nuclease activity of MobK-_6_His towards supercoiled DNA (scDNA) of plasmid pBGS-oriT1 containing pIGRK *oriT* (**c**) and empty vector pBGS18 (**d**). To estimate localization of individual DNA forms pBGS-oriT1 (**c**) and pBGS18 (d) scDNAs were digested using *EcoRI* (generation of linear form), and *NB.Bpu10I* (generation of open circle, oc form), ND—not digested DNA. Supercoiled plasmid DNA was incubated with MobK-_6_His or MobKY^179^F-_6_His in the presence (+) or absence (−) of Mg^2+^ divalent cations. (**e**,**f**) Comparative analysis of MobK-_6_His nuclease activity towards scDNA of: (**e**) pBGS-oriT8 containing minimal *oriT* (**f**) and pBGS-oriT9 containing oriT8 sequence with deletion of 29 bp.

Interactions of MobK, as well as MobKY^179^F with pIGRK DNA, were investigated by the electrophoretic mobility shift assay (EMSA) (Figure 3a). Recombinant MobK-_6_His and MobKY^179^F-_6_His proteins were purified and mixed with: (i) pIGRK DNA fragment containing 456-bp oriT1 (Figure 2a,c) as well as (ii) the control fragment—the pBGS18 plasmid MCS 136 bp sequence. EMSA demonstrated the presence of multiple shifts corresponding to complexes of both MobK-_6_His and MobKY^179^F-_6_His with oriT1. Only a weak MobK-control DNA fragment nucleoprotein complex formation (in the presence of high protein concentration) was visible.

### 2.4. MobK Forms Homodimers 

Mob proteins with two catalytic tyrosine residues (the one responsible for the initiation and the second responsible for the termination of RCR) are acting as monomers. Mobs with a single catalytic tyrosine have to form dimers to perform both DNA breaking reactions (during initiation and termination steps), as can be reviewed in [3]. Also, all known TR proteins act as dimers, as can be reviewed through an example in [28]. Therefore, it was decided to investigate MobK dimerization properties. A Chemical cross-linking experiment using glutaraldehyde (GA) as a cross-linker (Figure 3b) was performed. MobK-_6_His protein was incubated with increasing concentration of GA and analyzed by SDS-PAGE and Western-blot using anti-_6_His antibodies. In all analyzed samples, a signal corresponding to monomers of MobK-_6_His (~28 kDa) was visible (Figure 3b). In samples with a higher concentration of GA, the second band corresponding to MobK-_6_His dimers (~56 kDa) was also detected (Figure 3b). No additional bands of higher molecular size were observed, suggesting that MobK can form dimers but it is not able to oligomerize in solution.

### 2.5. MobKY^179^ Residue and Mg^2+^ Cations Are Critical for MobK Site-Specific Nuclease Activity

Conjugal relaxases as well as TR proteins exhibit topoisomerase-like site and strand-specific DNA nicking activity [3,20,28]. Using catalytic tyrosine residue –OH group as a nucleophile, they introduce a break in the DNA sequence [3,17]. To analyze MobK nuclease activity, purified recombinant proteins MobK-_6_His and mutant in predicted catalytic tyrosine MobKY^179^F-_6_His were used. Supercoiled DNA (scDNA) of pBGS-oriT1 (containing pIGRK *oriT* sequence) (Figure 2a,c) served as a substrate. Strong nicking activity of MobK-_6_His towards scDNA of pBGS-oriT1 was demonstrated (Figure 3c). After incubation with MobK-_6_His, almost whole scDNA was converted into open circle DNA (ocDNA). In contrast, no similar effect was observed when scDNA of pBGS-oriT1 was incubated with the same amount of mutated MobKY^179^F-_6_His protein (Figure 3c). Next, the specificity of MobK-_6_His nuclease activity was examined. For this purpose, MobK-_6_His was incubated with scDNA of empty vector pBGS18. In this control experiment, only a trace of topoisomerase-like activity was detected (Figure 3d). 

Divalent cations are indispensable cofactors for the nicking activity of “classical” Mobs [20,35]. They can also support the nicking activity of TR proteins [36]. In the conducted experiments, nuclease activities of recombinant MobK proteins in the presence or absence of Mg^2+^ divalent cations were analyzed. No DNA MobK-_6_His nicking activity was detected in the absence of Mg^2+^, suggesting strong MobK reliance on the presence of this cofactor (Figure 3c,d).

### 2.6. Mapping of pIGRK Minimal oriT Sequence

Our previous study revealed that a 456-bp intergenic region (here named oriT1), preceding *mobK* gene, contains minimal pIGRK *oriT* (Figure 2a,c) [27]. Now, using Clustal W [37], we aligned the oriT1 sequence with available sequences of minimal transfer origins from plasmid pCW3 (151 bp) and SGI1 IME element (125 bp) (Supplementary Figure S2), encoding related MOB modules [24,26]. Only some sequence similarity was identified between the central part of oriT1 and pCW3, as well as SGI1 *oriT*s. Subsequent studies on oriT1 DNA sequence led us to predict some putative functional elements (Figure 2c): (i) three different pairs of imperfect inverted repeats (IR1L and IR1R; IR2L and IR2R; IR3L and IR3R), (ii) two identical direct repeats (DR1-1 and DR1-2) (iii) as well as four short motifs 5′-AGCGA-3′ (two of them are located within DR1-1 and DR1-2, two others localize downstream from IR3R). Using BPROM [33], a putative *mobK* promoter (P*_mobK_*) was predicted. Just upstream from P*_mobK_*, a G-rich sequence was also identified. It was an intriguing observation, since the general GC content of pIGRK DNA is very low (33.4%) [27]. Predictions made with G4IPDB [34] showed that this sequence could form a secondary structure called G-quadruplex (G4). 

To identify the localization of minimal pIGRK transfer origin, conjugation experiments were performed. Initially, plasmid pRK-1 [32], pIGRK derivative with kanamycin resistance cassette (KmR) inserted into the central part of predicted the oriT1 (IR2L) (Figure 2a,c) was used. Previously analyzed pIGRK derivative, pIGRKKAN (with *kanR* gene cloned upstream from the *repR* gene and containing not disrupted oriT1 region) was efficiently transferred to recipient strain in the presence of an RP4 transfer system [27]. In the same experimental procedure, we analyzed pRK-1 mobilization properties. As a result, we observed that KmR cassette insertion completely inactivated pIGRK *oriT*, while pRK-1 were not able to mobilize for conjugal transfer (Figure 2b). 

In the next step, we analyzed the *oriT* activity of different pIGRK fragments (oriT1 shortened versions) cloned in pBGS18 in a two-plasmids system [25]. For this purpose, each putative functional elements, identified within the oriT1 sequence, was gradually deleted from the left (oriT2, oriT3, oriT4) and right (oriT5, oriT6, oriT7) end of oriT1 (Supplementary Figure S3). pBGS-oriT1 derivatives were efficiently transferred, even if they were deprived of IR1L and IR1R (pBGS-oriT2, pBGS-oriT3) as well as DR1-2 (pBGS-oriT5). Deletion of IR2L, similar to KmR insertion in pRK-1, results in *oriT* inactivation and pBGS-oriT4 was not able to transfer from the donor to the recipient cell. Deletion of G-rich sequence (oriT6) also results in *oriT* inactivation and pBGS-oriT6 was deprived of mobilization properties.

In this way, we demonstrated that 186 bp DNA fragment oriT8 (cloned in pBGS-oriT8), is a pIGRK minimal *oriT* sequence (Figure 2c and Supplementary Figure S3). 

G4 DNA structures are playing a significant role in the regulation of e.g., gene expression and DNA replication [38]. Recently, it was demonstrated that the presence of G4 within *oriT* can be critical for relaxosome recognition by T4CP [39]. For this reason, we decided to investigate whether the putative G4 sequence, located within oriT8, is necessary for *oriT* recognition and cleavage by the MobK or whether G4 plays a different role in *oriT* functioning. For this purpose, we performed an in vitro experiment to compare MobK-_6_His nicking activity towards scDNA of: (i) pBGS-oriT8 (containing minimal *oriT*) (ii) and pBGS-oriT9 (containing minimal *oriT* with deletion of 29 bp sequence containing putative G4 DNA) (Figure S3). As a result, we observed that MobK-_6_His nuclease activity was not affected by G4 sequence deletion. Both pBGS-oriT8 and pBGS-oriT9 scDNA were cleaved and converted to the ocDNA form to the same degree (Figure 3e,f).

## 3. Discussion

The TR-like conjugative relaxase family consists of three representatives: MobK from pIGRK plasmid, MpsA encoded by SGI1 IME element and TcpM from pCW3 plasmid. MobK is the first reported TR-like Mob [27]. However, so far it has not been studied in detail. Most recently, reported MpsA had only been poorly described [26]. For this reason, TcpM was the only representative of this unusual relaxase’s family that was studied in detail [24,25]. Here, we presented functional and molecular characterization of the pIGRK MobK protein as well as its cognate *oriT* sequence. 

We showed that tyrosine residue Y^179^, conserved among TRs and acting as a nucleophile in TR DNA processing, is indispensable for MobK activity. Substitution for this residue by phenylalanine results in a complete loss of MobK relaxase activity in vivo (Figure 2b). Using in vitro experiments, we demonstrated that Y^179^F mutation does not affect MobK DNA binding properties (Figure 3a), but causes loss of MobK nuclease activity (Figure 3c). In this study, the location of the minimal pIGRK *oriT* sequence was also determined. It is positioned within 186 bp DNA fragment, just upstream from the *mobK* gene promoter (Figure 2c). Investigating the specificity of MobK DNA binding and DNA processing properties, we showed that MobK specifically recognizes DNA containing *oriT*. However, weak and unspecific DNA binding and DNA cleavage were also detected (Figure 3a,d). We cannot exclude that some cellular proteins, present in recombinant MobK preps, could be responsible for observed unspecific interactions. DNA nicking activity towards scDNA lacking a cognate target *oriT* sequence was also indicated in the case of TcpM protein [24]. The authors speculated that TcpM could recognize DNA sequences with a partial identity to pCW3 *oriT*. Alternatively, additional accessory protein(s), interacting with TcpM, is (are) needed to increase TcpM (as well as pIGRK MobK) specificity [23,24,28]. 

The cross-linking experiment revealed that MobK can form dimers in solution (Figure 3b). This was also demonstrated in the case of TcpM protein [24]. However, TcpM additionally was able to form high-order complexes [24]. In our EMSA experiments, we demonstrated that MobK binds to the oriT1 sequence. Multiple shifts corresponding to DNA-MobK complexes were detected, which may suggest that MobK can form oligomers (at least in the presence of DNA) and/or there is more than one MobK binding site within oriT1 DNA. Some IR and DR sequences, putative MobK binding sites, were identified in oriT1. We cannot exclude that MobK interacts with oriT1 DNA as a relaxase (with minimal *oriT*) and as a regulatory protein, controlling its gene expression (with P*_mobK_*).

An interesting feature of MobK is the dependence on Mg^2+^ divalent cations. While divalent cations are obligatorily required by the representatives of whole Mob protein families described so far, it is known that DNA processing by TR enzymes occurs even without this cofactor [20,35]. However, most recently, it was unexpectedly demonstrated that the presence of Mg^2+^ is indispensable for the activity of PsrA recombinase, a TR enzyme from *Streptococcus pneumoniae* [36]. This finding supports our observation and proves that there is a certain group of TR enzymes (and TR-like proteins e.g., MobK) that are obligatorily dependent on divalent cations.

Mobs belonging to HUH and Rep_*trans* nucleases create a covalent bond with 5′-hydroxyl DNA end of the nicked strand. It allows the initiation of the rolling circle replication (RCR) from free 3′-OH end [3,20,23]. In the case of TR enzymes, the polarity of the DNA-protein covalent bond is the opposite. TRs, after DNA cleavage, remain bound to the 3′-OH end. In this way, RCR initiation is blocked [20]. Assuming that MobK is processing DNA similarly to TRs, the alternative mechanism (to RCR) should be acting to unwind plasmid transferred DNA strand and convert plasmid ssDNA to dsDNA in the donor cell. The simplest model describing MobK initiated CT could assume that DNA unwinding proceeds by unidentified host helicase and T4CP (motor protein) activity. ssDNA to dsDNA conversion is initiated from two single-strand replication origins present in each of the plasmid DNA strands (for initiation of replication in donor and recipient cell). However, so far only one functional single-strand origin was predicted in pIGRK (single strand initiation site, *ssi*) [32,40], located just downstream from the *mobK* gene (Figure 2a). 

The intriguing question is how the pIGRK MOB module interacts with not related RP4 T4SS transfer machinery. During bacterial conjugation, an *oriT* sequence is cleaved by relaxase and the DNA-Mob complex named relaxosome is formed [3]. Then, the relaxosome is recognized by cognate (or closely related) T4CP and targeted to the T4SS canal located in the donor cell envelope. The molecular basis of relaxosome recognition by the T4CP is not fully understood and has only been studied for a few model systems, as can be reviewed in [17]. Direct interactions between Mob (or DNA-Mob complex) and T4CP were reported (including RP4 TraG protein), suggesting the role of these interactions in relaxosome recognition [41,42]. It seems that also DNA structure (more than sequence) dependent direct DNA-T4CP interactions take place [43,44]. Recently, it was shown that R388 plasmid T4CP protein (TrwB) preferentially binds to G-quadruplex (G4) DNA. The presence of G4 DNA secondary structure also stimulates TrwB ATPase activity [32]. Interestingly, nonrelated FtsK-like conjugative translocase TraB from *Streptomyces venezuelae* plasmid pSVH1 also recognizes G4 DNA [45]. In the pIGRK minimal *oriT* sequence, putative G4 DNA was identified. Using in vivo and in vitro experiments, we proved that this sequence is indispensable for DNA transfer, but not for *oriT* DNA cleavage by MobK (Figure 3e,f and Supplementary Figure S3). Based on this, we speculate that MobK containing relaxosome can be recognized by RP4 TraG coupling protein through a G4 DNA signal. However, more complex studies should be conducted to verify this hypothesis.

A TR-like Mob proteins family only has three known representatives. Nonetheless, there are significant differences between their amino acid sequences and between nucleotide sequences of their cognate *oriT*’s (Figure 1, Supplementary Figure S2). This may suggest that TR-like Mobs are a diverse and numerous group of enzymes. In different bacterial species, the presence of proteins with TR-like DNA_BRE_C domains and (similar to TR-like Mobs) deprived of CB and AB domains have been reported [29]. Using BLASTP [46] and MobK sequence as a query, we indicated the presence of about 70 known similar proteins (80–100% sequence identity) with an orphan DNA_BRE_C domain designed as an integrase/recombinase. It becomes clear that more functional analysis should be performed before the correct biological role(s) of these proteins, as well as additional roles of TR-like Mobs, are stated. In this report, we demonstrated that MobK is a convenient model for studying the TR-like Mob proteins family and that further research on MobK will prove to be helpful in our better understanding of their biology.

## 4. Materials and Methods

### 4.1. Bacterial Strains, Plasmids and Culture Conditions

Bacterial strains and plasmids used in this study are listed in Supplementary Table S1. All strains were cultured in lysogeny broth LB medium (tryptone 10.0 g/L, yeast extract 5.0 g/L, and NaCl 5.0 g/L; pH 7.2–7.5) at 37 °C. When necessary, the medium was supplemented with appropriate antibiotics at the following concentrations: ampicillin (Ap)—100 μg/mL, kanamycin (Km)—25 μg/mL [for *E. coli* BL21(DE3)] or 50 μg/mL (for other strains), rifampicin (Rf)—50 μg/mL. 

### 4.2. DNA Manipulations

Plasmid DNA was isolated using a Plasmid Mini Isolation Kit (A&A Biotechnology, Gdańsk, Poland) according to the manufacturer’s instructions. DNA was introduced into bacterial cells by electroporation, using 1-mm gap cuvettes (BTX, San Diego, CA, USA) and a MicroPulse electroporator (Bio-Rad, Hercules, CA, USA), as described by Sambrook and Russell [47]. Details of plasmid constructions are presented in Supplementary Table S1. Routine DNA manipulations were carried out using standard procedures [47]. All restriction, DNA-modifying enzymes and DNA ligase were supplied by Thermo Fisher Scientific (Waltham, MA, USA). Amplification of DNA fragments by PCR was performed using Pfu or Taq DNA polymerase (Thermo Fisher Scientific, Waltham, MA, USA), appropriate primers and template DNAs. Point mutations in the *mobK* gene were generated using specific primers and a QuikChange Site-Directed Mutagenesis Kit according to the protocol supplied by the manufacturer (Stratagene, Santa Clara, CA, USA). All oligonucleotide primers used in this study are listed in Supplementary Table S2. Constructed plasmids were subjected to Sanger sequencing using 3130 Genetic Analyzer (Applied Biosystems, Foster City, CA, USA).

### 4.3. Western Blot Analysis

Protein samples were separated on standard 12% polyacrylamide SDS-PAGE gels. After electrophoresis, the gel was incubated in transfer buffer (1x SDS-PAGE buffer, 20% methanol) for 10 min. Nitrocellulose Blotting Membrane “Protran” 0.45 µm NC (Amersham, 10600002, Buckinghamshire, UK) was incubated in a transfer buffer for 10 min. Proteins were transferred from the gel onto the membrane using a VWR “PerfectBlue” Semi-Dry Electro Blotter Sedec M device (VWR, Radnor, PA, USA) with transfer buffer at 55 mA (1 mA/cm^2^) for 90 min. The transfer membrane was then bathed for 30 min in blocking buffer (20 mM Tris base, 150 mM NaCl, 0.05% Tween 20.5% nonfat dried milk; pH 7.5) at room temperature. The blocked membrane was incubated with a 1:2,000 diluted mouse anti-His tag antibody (Thermo Fisher Scientific, MA1-21315-1MG, Waltham, MA, USA) for 2 h at room temperature. Next, the membrane was washed 3 times for 20 min in washing buffer (20 mM Tris base, 150 mM NaCl, 0.05% Tween 2; pH 7.5). The membrane was then incubated with 1:10,000 diluted anti-mouse IgG (H-L) AP-conjugated antibody (Promega, S3721, Madison, WI, USA) for 1 h at room temperature. After a further two 5 min washes, immunoreactive bands on the blot were detected using the BCIP/NBT (5-bromo-4-chloro-3-indolyl-phosphate/nitro blue tetrazolium) Color Development Substrate (Promega, S3771, Madison, WI, USA), according to the manufacturer’s instructions.

### 4.4. Overexpression and Purification of _6_His-Tagged MobK and MobKY^179^F Proteins

The *mobK* and *mobK*Y^179^F gene variants of pIGRK were cloned in expression vector pET28b+ (Supplementary Figure S1 and Table S1). *E. coli* BL21(DE3) strains harboring each construct were cultured overnight in an LB medium supplemented with kanamycin at 37 °C with shaking (180 rpm). For protein overexpression, 8 mL of the overnight cultures were added to 1000 mL of fresh LB + kanamycin medium, and incubation was continued at 37 °C with shaking (180 rpm). When the culture reached an OD600 of 0.4, isopropyl β-D-1-thiogalactopyranoside (IPTG) was added to a final concentration of 0.4 mM to induce the expression of the _6_His-tagged proteins. The cultures were then further incubated for 12 h at 25 °C with shaking (100 rpm). The cells were collected by centrifugation (15 min, 6500× *g*, 4 °C) and resuspended in 15 mL of lysis buffer: 50 mM Tris-HCl pH 8.0, 500 mM NaCl, 20 mM imidazole and 300 μL of 100 mg/mL lysozyme, supplemented with 1 mM PMSF and Protease Inhibitor Cocktail (Sigma, Hilden, Germany). After holding on ice for 15 min, the cells were disrupted by sonication and the obtained lysates were centrifuged (30 min, 22,000× *g*, 4 °C) to pellet cell debris. All subsequent steps were performed at 4 °C. The cleared lysates were incubated with 0.5 mL of Ni-NTA beads (Qiagen, Steinheim, Germany) for 60 min, with gentle shaking. The Ni-NTA resin was then given a series of washes: (i) twice with 8 mL of W1 buffer (50 mM Tris-HCl pH 8.0, 500 mM NaCl, 20 mM imidazole, 0.5 mM EDTA 2 mM DTT), (ii) once with 4 mL of W2 buffer (50 mM Tris-HCl pH 8.0, 2 M NaCl, 20 mM imidazole, 0.5 mM EDTA 2 mM DTT). The _6_His-tagged proteins were finally eluted with 3 portions of 0.5 mL buffer W1 (with increasing concentration of imidazole: 50, 100 and 150 mM, respectively). Purified proteins were dialyzed against buffer D (50 mM Tris-HCl pH 8, 500 mM NaCl, 0.5 mM EDTA, 2 mM DTT) and concentrated using ultrafiltration tubes Vivaspin 6 MWCO 10,000 (GE Healthcare, GE28-9322-96, Piscataway, NJ, USA). The final concentration of the purified recombinant proteins was estimated using the Bradford dye-binding method. All proteins were analyzed by SDS-PAGE, see Supplementary Figure S4 and MALDI-TOF mass spectrometry to confirm their identity. Additional protein bands (marked by an asterisk) correspond to *E. coli* SlyD protein (identified by trypsin digestion and MALDI-TOF mass spectrometry) persistent contaminant of _6_His-tagged recombinant proteins purified by metal affinity chromatography [48]. Protein aliquots were frozen in liquid nitrogen and stored at −70 °C.

### 4.5. Electrophoretic Mobility Shift Assay

Using pBGS-oriT1 plasmid (Supplementary Table S1) construct as a template, the cloned pIGRK DNA fragment was amplified by PCR with a “universal” M13 forward and reverse primers—M13pUCf and FAM-labeled M13pUCrFAM (oligos 7 and 8 in Supplementary Table S2) Obtained PCR product was subsequently purified using a Clean-Up kit (A&A Biotechnology, Gdańsk, Poland). The same primer pair was used for the amplification of a 136 bp DNA fragment of pBGS18, which served as a negative control. 

Binding reactions of 20 μL containing 0.6 pmol of oriT1 or 1.2 pmol of control FAM-labeled DNA fragments, 1× binding buffer (25 mM Tris-HCl pH 7.6, 15 mM MgCl_2_, 1 mM DTT, 0.1 mM EDTA, 80 µg/mL BSA) were incubated for 30 min at 25 °C with an increasing amount of MobK-_6_His or MobKY^179^F-_6_His (0, 16, 24, 32, 40, 48 pmol of protein). After incubation, reactions were gently mixed with 6 μL of 50% glycerol and loaded on a 1.75% agarose gel cast with 0.5× TBE buffer. Protein-DNA complexes were then separated by electrophoresis in 0.5× TBE buffer at 5 V/cm at room temperature and DNA fragments were visualized using an Imager 600 (Amersham, Buckinghamshire, UK).

### 4.6. Bacterial Mating Procedure

The experiment was performed according to the procedure described previously [27]. The mating procedure was performed in liquid medium using *E. coli* S17-1 [49] carrying a kanamycin-resistant plasmid (pBGS-oriT [1,2,3,4,5,6,7,8,9] or pRK-1) (Supplementary Figures S1 and S3 and Table S1), as the donor strain and rifampicin-resistant *E. coli* DH5αR [50] (as the recipient). In transfer experiments with pBGS-oriT (harboring only *oriT* sequence without *mobK* relaxase gene) additional plasmid, pWSK29 [51] derivatives (pWSK-1/2/3) (Supplementary Figure S1 and Table S1) were used as a MobK (or as MobK mutants) source in donor strain. The mating mixture was incubated for 2 h at 37 °C (without agitation). The cell suspension was then diluted, and 100 μL of appropriate dilutions were plated on selective media containing rifampicin and kanamycin to select for transconjugants. Spontaneous resistance of the recipient strains to the antibiotics used in selection was not observed under these experimental conditions. The plasmid content of transconjugants was verified by screening several colonies using a rapid alkaline extraction procedure and agarose gel electrophoresis. All matings were repeated at least three times. As control of conjugation experiment, *E. coli* S17-1 strains harboring pRK415 [52] (positive control) and empty pBGS18 [53] (negative control) were used (Supplementary Table S1).

### 4.7. In Vitro MobK Nuclease Activity Assay

Isolation of DNA substrates (supercoiled, scDNA of pBGS18 and pBGS-oriT1 as well as pBGS-oriT8 and pBGS-oriT9 plasmids) (Supplementary Figure S3 and Table S1) was performed using Plasmid Midi Isolation Kit (A&A Biotechnology, Gdańsk, Poland) according to the manufacturer’s instruction with some modifications. All steps of DNA isolation (except L2 adding and incubation with L2 buffer) were performed at 4 °C. Nuclease-free water was added to the obtained DNA pellet and DNA was dissolved overnight at 4 °C, while scDNA was stored at 4 °C.

Nuclease activity was determined according to the procedure described by Rozhon and coworkers [54] with some modifications. A total of 1 µg of scDNA sample was incubated with 400 ng of MobK-_6_His or MobKY^179^F-_6_His at 37 °C for 90 min in a reaction mixture containing: 25 mM Tris-HCl pH 7.6, 1 mM DTT, 0.1 mM EDTA and with or without of 5 mM Mg^2+^. Reactions were stopped by adding 5 µL of stop buffer (2 mg/mL proteinase K, 2% SDS, 100 mM EDTA, 100 mM Tris-HCl pH 8.0, 20% glycerol and 0.05% bromophenol blue) and prolonging incubation at 37 °C for 20 min. The samples were directly loaded onto a 1% agarose gel and run in 0.5 x TBE buffer. DNA was visualized by gel staining in GelRed Nucleic Acid Gel Stain (Biotum, 41001, Fremont, CA, USA) and using an Imaginer 600 device (Amersham, Buckinghamshire, UK).

### 4.8. Glutaraldehyde Cross-Linking

Glutaraldehyde (GA) cross-linking experiment was performed according to the procedure described previously [32]. The formation of MobK-_6_His dimers was investigated by incubation of approximately 400 ng of protein with increasing concentration of GA (final concentration: 0%, 0.001%, 0.0025%, 0.005%, 0.025%, 0.05%). Protein samples were separated by SDS-PAGE and _6_His- tagged MobK protein was identified by Western blotting.

### 4.9. Bioinformatics Analyses

DNA and protein sequences were aligned using BLAST search tool (https://blast.ncbi.nlm.nih.gov/Blast.cgi) (accessed on 9 March 2021) [46] or Clustal W (https://www.genome.jp/tools-bin/clustalw) (accessed on 15 Feburary 2021) [37]. Protein secondary structures were determined using JPred4 (http://www.compbio.dundee.ac.uk/jpred4) (accessed on 22 Feburary 2021) [30]. Molecular masses and isoelectric points of proteins as well as *mobK* promoter were predicted using Compute pI/Mw and BPROM tools respectively, available through the ExPASy server (https://web.expasy.org) (accessed on 17 March 2020) [33]. Putative G-quadruplex forming G-rich nucleotide sequence was predicted using G4IPDB (http://bsbe.iiti.ac.in/bsbe/ipdb/index.php) (accessed on 9 March 2021) [34]. DNA sequences analyzed in this study: pIGRK (acc. no. AY543071.1), pCW3 (acc. no. DQ36603.1) and SGI1 (acc. no. AF261825.1). Protein sequences analyzed in this study: MobK (acc. no. AAS55463.1), TcpM (acc. no. ABC96296.1), MpsA (acc no. AAK02039.1) and MpsB (acc. no. AAK38397.1).

## Data Availability

Not applicable.

## Supplementary Materials

The following are available online at https://www.mdpi.com/article/10.3390/ijms22105152/s1, Figure S1: Construction of plasmids expressing *mobK* gene and its derivatives, Figure S2: Alignments of the pIGRK oriT1 region and minimal *oriT*s from SGI1 and pCW3, Figure S3: Mapping of pIGRK minimal *oriT*, Figure S4: SDS-PAGE of recombinant proteins purification procedure, Table S1: Bacterial strains and plasmids used in this study, Table S2: Sequences of oligonucleotides used in this study.
