# Supplementary material for: Molecular and Functional Characterization of MobK Protein—A Novel-Type Relaxase Involved in Mobilization for Conjugational Transfer of Klebsiella pneumoniae Plasmid pIGRK
Source: Int J Mol Sci. 2021 May 13;22(10):5152. doi: 10.3390/ijms22105152 (PMC8152469; doi:10.3390/ijms22105152)
Supplement: Supplementary file 1 [file ijms-22-05152-s001.zip › ijms-1209396-supplementary.pdf]

Supplementary Materials

Molecular and functional characterization of MobK – a novel-type relaxase involved in mobilization for conjugational transfer of *Klebsiella pneumoniae* plasmid pIGRK

Table S1. Bacterial strains and plasmids used in this study

Bacterial strain	Description	
E. coli **DH5α**	F^- *endA1 glnV44 thi-1 recA1 relA1 gyrA96 deoR nupG* Φ80d *lacZ*ΔM15 Δ(*lacZYA-argF*)*U169*, *hsdR17* (r_K^- m_K^+), λ	Invitrogen
E. coli **DH5αR**	Rifampicin resistant mutant of DH5α strain	[50]
E. coli **BL21(DE3)**	F^- *ompT gal dcm lon hsdS*$_B$ (r_B^- m_B^-) λ(*DE3*) *pLysS*(Cm^R)	Invitrogen
E. coli **S17-1**	hsdR recA proRP4-2 (Tc::Mu; Km::Tn7)(λpir)	[49]
**Plasmid**	**Description**	
**pIGRK**	2348 bp cryptic plasmid isolated from *Klebsiella pneumoniae* 287-w	[3]
**pET28b+**	Km^R, *ori* pBR, *ori* F1, plasmid vector for $_6$His tagged proteins overexpression	Invitrogen
**pET-mobK**	pET28b+ with PCR amplified *mobK* gene from pRK-1 (using 9 and 10 oligo, Table S2) (1251-1976 bp pIGRK) cloned in *NcoI* and *XhoI* sites	this study
**pET-mobKYF**	pET28b+ with PCR amplified *mobK*Y^{179}F gene from pRK-1_14 (using 9 and 10 oligo, Table S2) cloned in *NcoI* and *XhoI* sites	this study
**pBGS18**	Km^R; *oriV* pMB1; cloning vector	[53]
**pBGS-oriT1**	pBGS18 carrying a DNA fragment of pIGRK (positions 806–1261) containing *oriT*, renamed from pBGS18/3oriT	[3]
**pBGS-oriT2**	pBGS18 with PCR amplified fragment of pIGRK (positions 959–1261) (using 11 and 14 oligo, Table S2) cloned in *XbaI* and *SmaI* sites	this study
**pBGS-oriT3**	pBGS18 with PCR amplified fragment of pIGRK (positions 994–1261) (using 12 and 14 oligo, Table S2) cloned in *XbaI* and *SmaI* sites	this study
**pBGS-oriT4**	pBGS18 with PCR amplified fragment of pIGRK (positions 1035–1261) (using 14 and 15 oligo, Table S2) cloned in *XbaI* and *SmaI* sites	this study
**pBGS-oriT5**	pBGS18 with PCR amplified fragment of pIGRK (positions 806–1179) (using 13 and 17 oligo, Table S2) cloned in *SmaI* and *HindIII* sites	this study
**pBGS-oriT6**	pBGS18 with PCR amplified fragment of pIGRK (positions 806–1151) (using 13 and 18 oligo, Table S2) cloned in *SmaI* and *HindIII* sites	this study
**pBGS-oriT7**	pBGS18 with PCR amplified fragment of pIGRK (positions 806–1132) (using 13 and 19 oligo, Table S2) cloned in *SmaI* and *HindIII* sites	this study
**pBGS-oriT8**	pBGS18 with PCR amplified fragment of pIGRK (positions 994–1179) (using 16 and 17 oligo, Table S2) cloned in *XbaI* and *HindIII* sites	this study
**pBGS-oriT9**	pBGS18 with PCR amplified fragment of pIGRK (positions 994–1151) (using 16 and 18 oligo, Table S2) cloned in *XbaI* and *HindIII* sites	this study
**pWSK29**	Ap^R; *oriV* pSC101; cloning vector	[51]
**pWSK-1**	pWSK29 carrying *mobK* gene with its own promoter P_{mobK}, renamed from pWSK-int	[3]
**pWSK-2**	pWSK-1 with mutation in 179 codon of *mobK* (Y^{179}F mutation), pIGRK insert replaced by the corresponding fragment of pRK-1_14 (PCR amplified using 5 and 6 oligo, Table S2)	this study
**pWSK-3**	pWSK-1 with insertion of 20 bp sequence (1328_1329insGGATCCTAGAGGATCCGCAC) in *mobK* performed by PCR amplification (using 1 and 2 oligo, Table S2)	this study
**pRK-1_14**	pRK-1 with a mutation in 179 codon of *mobK* (Y^{179}F mutation, 1786A>T) introduced by PCR amplification (using 5 and 6 oligo, Table S2)	this study
**pRK-1**	Km^R derivative of pIGRK, KmR cassette incorporated downstream from the replication initiator gene, within *oriT* (1024_1028insKmR) in the place of three nucleotides (TTG, 1025_1027del)	[6]
**pRK415**	Tc^R; *oriV* RK2; *oriT* RK2	[52]

The introduced mutations are described according to the following scheme: (i) deletions: 000_000del (nucleotide position of the first deleted pair of bases _ nucleotide position of the last deleted pair of bases, deletion), (ii) insertions: 000_000insXYZ (nucleotide position of the first pair of bases above the insertion position _ the first pair of bases below the insertion, ins - insertion; XYZ - the name of the inserted element), (iii) nucleotide substitutions: 000X>Y (000X - position in the sequence and nucleotide occurring in the sequence originally, Y - the nucleotide introduced in its place), (iv) amino acid substitutions: $X^{00}Y$ (X - original amino acid, ^{00} - position in the sequence, Y - introduced amino acid). Sequence coordinates, unless otherwise stated, pIGRK (GenBank: AY543071.1).

Table S2. Sequences of oligonucleotides used in this study

	Name	Oligonucleotide sequence (5′→3′)
**1**	RK_W_F	GA**GGATCC**GCACAATCTAAAATGTGCTAAC
**2**	RK_W_R	TA**GGATCC**GTGCGATCGCATAATC
**3**	RK2G	GAGGA**GAATTC**GCGAAGGCCATAAAATTGCCA
**4**	RK2D	AAAAA**TCTAGA**ACCATCCAGTTACCCGTTCC
**5**	MobKYF_F	CTCAGTTAGATCTCAGCAGAGT**ttc**GGAAGAAAGAGC
**6**	MoBKYF_R	GCTCTTTCTTCC**gaa**ACTCTGCTGAGATCTAACTGAG
**7**	M13pUCf	CCAGTCACGACGTTGTAAAACG
**8**	M13pUCrFAM*	FAM-AGCGGATAACAATTTCACACAGG
**9**	pETmobKF	GCCT**CCATGG**GTAATTCAAAAAGAAATATAAAAAAACTAAATG
**10**	pETmobKR	ATT**CTCGAG**CATTTTGAAGCGACGAACGGG
**11**	XRKORITF	ATT**TCTAGA**CATTGTTTCTCCACATTTGC
**12**	HRKORITR	TAGAT**AAGCTT**TGCTCTATCCCTAAAATG
**13**	Int5 [3]	GAAATCTCGAAAGAATGGAAGGAAAAG
**14**	Int6 [3]	GAATTACCCATATTGATTTTTCCTCA
**15**	ORIT2F	TAA**TCTAGA**CCGTCTTTTTGGGTGGAAC
**16**	ORIT3F	TAA**TCTAGA**TTCCACCCGGATATAACAG
**17**	ORITR1R	TAT**AAGCTT**CCGTAAAACCCAAACCCTC
**18**	ORIT5R	TAT**AAGCTT**GTTCAATTTTTCGCTATCGCTC
**19**	ORITR2R	TAT**AAGCTT**CTCAAATCGAACAACGACC

The underlined bolded fragments of the sequences indicate sites recognized by restriction enzymes attached to oligonucleotides. Positions of the changed nucleotides, introduced mutations, are highlighted by small bolded fonts. *FAM (fluorescein amidite) attached to the 5′-end of the oligonucleotide.

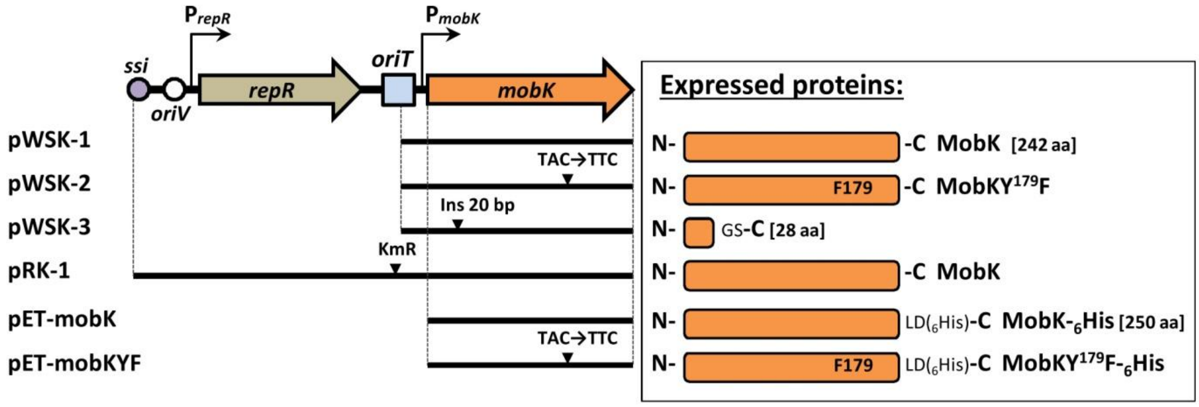

(a) (b)

Figure S1. Construction of plasmids expressing *mobK* gene and its derivatives. **(a)** Schematic representation of parental pIGRK plasmid and constructed vectors. Black solid lines represent DNA fragments of pIGRK used in plasmid construction. The insertion sites for the kanamycin resistance cassette (KmR), the substitution of tyrosine codon to phenylalanine codon as well as insertion of 20 bp in *mobK* gene are indicated by black triangles. *oriV* – vegetative replication origin, *ssi* – single strand initiation, **(b)** schematic representation of proteins expressed from constructed vectors: MobK (native protein), MobKY^{179}F (MobK with substitution of catalytic tyrosine to phenylalanine) as well as 26 *N*- terminal aa of MobK with two amino acids (GS) attached to its *C*-terminus (result of disruption of *mobK* gene by insertion of 20 bp DNA fragment in *mobK* ORF), MobK-$_6$His and MobKY^{179}F-$_6$His recombinant proteins used in *in vitro* experiments.

(a)

```
pIGRK         GAAATCTCGAAAGAATGGAAGGAAAAGTTTAAAGACGAAGAGGAAAATTTCGAATTTGGT
SGI1          ------------------------------------------------------------
pIGRK         TTTGAATCGGAAATATAAAACCGCCCTCGCCGGGCAGGCGAATCCCTTATTGAAATAGAA
SGI1          ------------------------------------------------------------
pIGRK         TAAATTCTATTCCACTAAGGGATTTTTTTTATTCATTGTTTCTCCACATTTGCAATATTG
SGI1          --------------------------------GTATAATTCGCGCACATTCGTGCGCGGT
                                                **  **    ****** *

pIGRK         ACATTAACTTCCACCCGGATATAACAGTAGTATAAGTTGTTGTTTCAACCCGTCTTTTTG
SGI1          GCGAAAGCCTAGAGCCCTTGAGGCTCAAGGCTTCCGTCGGGGGCTCTACCCCCGTCTCTG
               *   * * *  * **    *        *  *  ** *  *  ** ****   * * **

pIGRK         GGTGGAACAACAA-GGCATTTTAGGGATAGAGCAAAGCGAAGGCCATAAAATTGCCACCC
SGI1          TTTACGCCTACGGCGACAGAGACGGGGTGGAGCATAG-----------------------
                *    * **   * **     *** * ***** **

pIGRK         CCAACCGGGGGTCGTTGTTCGATTTGAGCGATAGCGAAAAATTGAACATAAGGGGGGAGG
SGI1          ------------------------------------------------------------
pIGRK         GTTTGGGTTTTACGGTATTTCAAATTTGAGCAAAGCGAATTTTTGAAATTTCCGGTTCTT
SGI1          ------------------------------------------------------------
pIGRK         TTAATTTGCAATGAGGAAAAATCAATATGGGTAATTC
SGI1          -------------------------------------
```

(b)

```
pIGRK         GAAATCTCGAAAGAATGGAAGGAAAAGTTTAAAGACGAAGAGGAAAATTTCGAATTTGGT
pCW3          ------------------------------------------------------------
pIGRK         TTTGAATCGGAAATATAAAACCGCCCTCGCCGGGCAGGCGAATCCCTTATTGAAATAGAA
pCW3          ------------------------------------AAGGAACTTTACAGGGAACTTTAA
                                                     ***      *  *** *  **

pIGRK         TAAATTCTATTCCACTAAGGGATTTTTTTTATT--CATTGTTTCTCCACATTTGCAATAT
pCW3          -AAATTTAAATTGATATAAAAGTTCCCTGTATTAGTATAAGTATTTTAAAGGTATA-TAT
               *****  * *  *   *    **   * ****   **   *  *  * *  *  * ***

pIGRK         TGACATTAACTTCCACCCGGATATAACAGTAGTATAAGTTGTTGTTTCAACCCGTCTTTT
pCW3          CATTATTAGTTCCTTATCG--TATTATAG-AGTATATATTATATATATAATATATACATA
                  ****  * *    **  *** * ** ******  ** *   *  **    *   *

pIGRK         TGGGTGGAACAACAAGGCATTTTAGGGATAGAGCAAAGCGAAGGCCATAAAATTGCCACC
pCW3          TAA-TGTATTGG------------------------------------------------
              *   ** *

pIGRK         CCCAACCGGGGGTCGTTGTTCGATTTGAGCGATAGCGAAAAATTGAACATAAGGGGGGAG
pCW3          ------------------------------------------------------------
pIGRK         GGTTTGGGTTTTACGGTATTTCAAATTTGAGCAAAGCGAATTTTTGAAATTTCCGGTTCT
pCW3          ------------------------------------------------------------
pIGRK         TTTAATTTGCAATGAGGAAAAATCAATATGGGTAATTC
pCW3          --------------------------------------
```

Figure S2. Alignments of pIGRK oriT1 region and minimal *oriT*s from SGI1 and pCW3. **(a)** oriT1 *vs* SGI1 *oriT* sequence. **(b)** oriT1 *vs* pCW3 *oriT* sequence. pIGRK minimal *oriT* sequence is underlined.

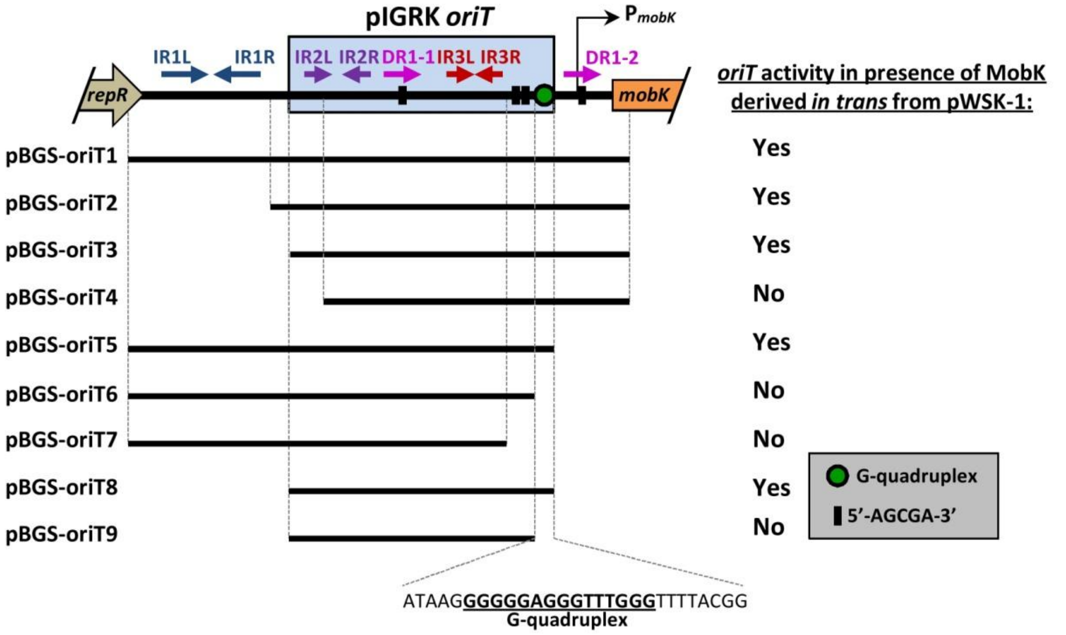

Figure S3. Mapping of pIGRK minimal *oriT*.

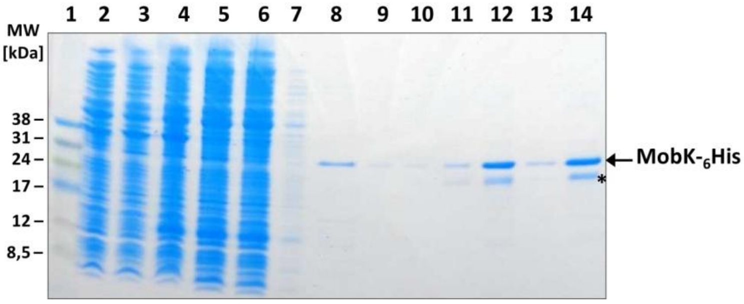

(a)

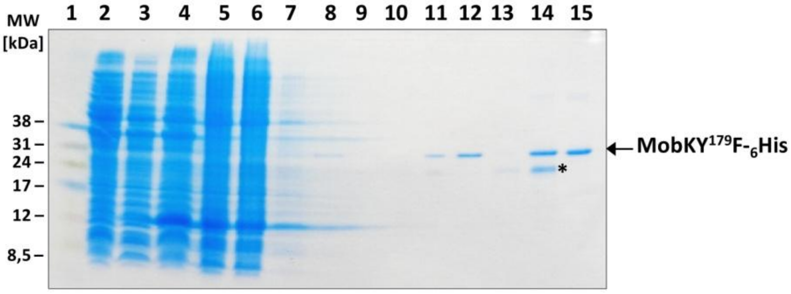

(b)

Figure S4. SDS-PAGE of recombinant proteins purification procedure. **(a)** MobK-$_6$His purification, **(b)** MobKY^{179}F-$_6$His purification. Lanes: (1) - protein marker, (2) - bacterial culture before induction, (3) - bacterial culture after induction, (4) - bacterial pellet of centrifuged lysate, (5) - supernatant of centrifuged bacterial lysate, (6) - proteins not attached to the Ni-NTA resin, (7, 8 and 9) - washes, (10, 11 and 12) - elutions with increasing concentration of imidazole: 50, 100 and 150 mM respectively, (13-15) - concentrated elution fractions: (a) wash 11 lane (13), wash 12 lane (14); (b) wash 11 lane (14), wash 12 lane (15). Protein bands marked by asterisk correspond to *E. coli* SlyD protein (identified by trypsin digestion and MALDI-TOF mass spectrometry) persistent contaminant of $_6$His-tagged recombinant proteins purified by metal affinity chromatography [48].
